# Efficacy of the Combination of Teriparatide and Denosumab in the Treatment of Postmenopausal Osteoporosis: A Meta-Analysis

**DOI:** 10.3389/fphar.2022.888208

**Published:** 2022-05-24

**Authors:** Yang Sun, Yue Li, Jiangbi Li, Xiaoping Xie, Feng Gu, Zhenjiang Sui, Ke Zhang, Tiecheng Yu

**Affiliations:** ^1^ Department of Orthopedics, The First Hospital of Jilin University, Jilin Changchun, China; ^2^ Department of Social Psychiatry, The Affiliated Brain Hospital of Guangzhou Medical University, Guangzhou, China

**Keywords:** postmenopausal osteoporosis, combination treatment, teriparatide, denosumab, monotherapy

## Abstract

**Aim:** Evidence on the efficacy of combination treatment of teriparatide and denosumab for osteoporosis remains controversial. We aim to compare the efficacy between the combination treatment and monotherapy among patients with postmenopausal osteoporosis.

**Methods and results:** We systematically searched PubMed, EMBASE, the Cochrane Library, and Web of Science up to 26 January 2022, for relevant studies. This meta-analysis reviewed all randomized controlled trials (RCTs) that reported on the combination treatment of teriparatide and denosumab in patients with postmenopausal osteoporosis. The articles were examined individually by two reviewers, and the relevant data was extracted. We combined weighted mean difference (WMD) for bone mineral density (BMD) using random- or fixed- effect models and conducted subgroup analyses. Sensitivity analyses were performed, and possible publication bias was also assessed. Overall, combination treatment enhanced the mean percent change of bone mineral density in lumbar spine than monotherapy (WMD = 2.91, 95%CI: 1.983.83; *p* = 0.00). And, combination treatment has been beneficial for enhancing the mean percent change of BMD in hip (WMD = 3.19, 95%CI: 2.25∼4.13; *p* = 0.00). There was no significant difference between combination treatment and monotherapy in terms of the adverse events (RR = 0.81, 95%CI: 0.45∼1.45; *p* = 0.472).

**Conclusion:** The meta-analysis indicates that combination treatment led to greater BMD at the lumbar spine and hip in comparison to monotherapy, without an increased incidence of adverse events.

**Systematic Review Registration:** (https://inplasy.com/), identifier (Inplasy Protocol 2734).

## 1 Introduction

The most prevalent bone disease among aged women is postmenopausal osteoporosis (Kahwati et al., 2018). It is a major cause of fracture, which results in substantial morbidity, mortality, and financial burdens (Liu Y. et al., 2018; Nayak and Greenspan, 2018). Skeletal fragility and microarchitectural degeneration are hallmarks of the disease (Black and Rosen, 2016a). The most of patients with postmenopausal osteoporosis are probably attributable to genetically determined low bone mass combined with bone loss associated with oestrogen deficiency (Andreopoulou and Bockman, 2015). In the United States, osteoporosis causes millions of fractures each year, the vast majority of which occur in postmenopausal women (Black and Rosen, 2016a). Moreover, nearly 9 million osteoporosis-related fractures occur each year (Liu GF. et al., 2018)*.* The global cost of osteoporosis-related fractures is supposed to surpass $25.3 billion per year by 2025 (Burge et al., 2007).

Until now, a variety of pharmacotherapies have been available for postmenopausal osteoporosis (Khosla and Hofbauer, 2017). Postmenopausal osteoporosis medications could generally be divided into two categories. Antiresorptive medications like nitrogen-containing bisphosphonates and denosumab, a receptor activator of nuclear factor B ligand inhibitor, are the most commonly used drugs. The other category is the anabolic agents teriparatide [PTH-(1–34)] and PTH [PTH-(1–84)], which are mostly used for patients with serious and established osteoporosis. In addition to stimulating osteoblastic bone formation, these peptides also stimulate bone resorption (Dempster et al., 2012). Despite the fact that therapeutic options for postmenopausal osteoporosis have increased during the past few years (Khosla and Hofbauer, 2017), no currently approved therapy seems to restore normal bone integrity in the most of patients with established osteoporosis and choices for severe patients are still constrained. Approved osteoporosis therapies are generally constrained to the prescription of a single drug with a set dosage and dosing frequency. Combination treatment with anabolic and antiresorptive agents has been suggested as a method to improve the treatment efficacy. However, attempts to combine teriparatide or PTH with bisphosphonates have failed since no combination has ever been proven to be consistently better than monotherapy (Black et al., 2003; Finkelstein et al., 2003; Finkelstein et al., 2010; Cosman et al., 2011). With the publication of clinical trials and the accumulation of clinical experience, we have found that the combination of teriparatide and denosumab probably has better efficacy in postmenopausal osteoporosis (Tsai et al., 2013; Leder et al., 2015; Idolazzi et al., 2016; Nakamura et al., 2017; Suzuki et al., 2019). To further corroborate the clinical value of the combination treatment, we have made this meta-analysis to provide the basis for this new treatment modality.

## 2 Methods

The Preferred Reporting Items for Systematic Reviews and Meta-Analysis (PRISMA) statement is followed in this study (Moher et al., 2009). A formal protocol was developed and registered on the INPLASY international platform of registered systematic review and meta-analysis protocols (INPLASY Protocol 202210092).

### 2.1 Literature Search Selection Criteria

Two independent reviewers (Sun and Li) systematically scanned PubMed, EMBASE, Cochrane Library and Web of Science for related articles published up to 26 January 2022. Medical Subject Headings (MeSH) terms and Keywords were used for searching databases. The search terms include: “Osteoporosis, Postmenopausal,” “Perimenopausal Bone Loss,” “Bone Loss, Postmenopausal,” “Bone Losses, Postmenopausal,” “Postmenopausal Bone Losses,” “Osteoporosis, Post-Menopausal,” “Osteoporoses, Post-Menopausal,” “Osteoporosis, Post Menopausal,” “Post-Menopausal Osteoporoses,” “Post-Menopausal Osteoporosis,” “Postmenopausal Osteoporosis,” “Osteoporoses, Postmenopausal,” “Postmenopausal Osteoporoses,” “Bone Loss, Perimenopausal,” “Bone Losses, Perimenopausal,” “Perimenopausal Bone Losses,” “Postmenopausal Bone Loss,” “Teriparatide,” “hPTH (1–34),” “Human Parathyroid Hormone (1–34),” “Parathar,” “Teriparatide Acetate,” “Forteo,” “Denosumab,” “Xgeva,” “AMG 162,” “Prolia”. In addition, RCTs that were registered as completed but not yet published were searched on ClinicalTrials.gov (http://www.clinicaltrials.gov). By examining titles and abstracts, two investigators individually screened the literature. We examined the full text to complete the assessment when the information from the titles and abstracts was insufficient to decide whether to include or exclude the studies. A full discussion between the two investigators was held to resolve any disagreements in the study screening process, and a third investigators was advised if a consensus could not be achieved. Eligible articles were selected if they fulfilled the listed criteria: 1) Population: Postmenopausal osteoporosis; 2) intervention: combination treatment of teriparatide and denosumab; 3) comparison: Monotherapy (teriparatide or denosumab); 4) outcome: Percentage change at end of study in the areal BMD in the lumbar spine or total hip, percentage change at end of study in the serum 25(OH)D; 5) design: RCT. We excluded the articles by the listed exclusive criteria: 1) Study not available in full; 2) low sample size (≤3 subjects/group); 3) duplicated articles; 4) animal experiments; 5) subjects had symptom of vitamin D deficiency (serum level less than 20 ng/ml), hyperparathyroidism, estrogen application, other acquired or congenital bone disease.

### 2.2 Data Extraction and Quality Assessment

Two reviewers extracted the data using a pre-designed extraction form. The collected data contains name of the first author, publication date, country, sample size, mean age, interval and dose, adjuvant, outcome, and other baseline clinical characteristics. Data extraction disagreements were resolved by discussions among the investigators, with the assistance of a third investigator if necessary. Then, two investigators individually assessed the quality of the included articles based on the Cochrane risk-of-bias tool. Random sequence generation; allocation concealment; blinding of participants and personnel to the study protocol; blinding of outcome assessment; incomplete outcome data; selective reporting; and other bias were all evaluated in each trial and given a high, low, or unclear risk of bias score.

### 2.3 Statistical Analysis

The mean percent change of BMD in postmenopausal osteoporosis patients receiving prescriptions for combination treatment or monotherapy is our primary outcome. The pooled results were calculated using the weighted mean difference (WMD) with a 95% confidence interval. *p* < 0.05 indicates statistically significant. In addition to this, we recorded the number of adverse events in each group in each trial and calculated the risk ratio (RR) with 95% CI by Stata. The Q-statistic test was used to assess the heterogeneity between studies. *p* < 0.10 for the Q-statistic indicates statistically significance (Higgins et al., 2003). The I^2^ index was used to determine the degree of inconsistency. It is sorted as unimportant heterogeneity (I^2^ ≤ 40%), moderate to substantial heterogeneity (I^2^ >40% and <75%), or considerable heterogeneity (I^2^ ≥ 75%) (Higgins et al., 2003). Depending on the heterogeneity between studies, results were pooled using a random- or fixed-effect model. Subgroup analyses could be conducted according to participant characteristics, like country. Before conducting subgroup analyses, tests can be conducted using regression to determine whether the characteristic being examined affects the heterogeneity of the final results. Moreover, owing to the differences in interventions of the control, subgroup analyses were also performed based on the interventions of the control, regardless of the degree of heterogeneity. To examine the robustness of the outcomes, sensitivity analyses were conducted by removing each selected study, and publication bias was tested by funnel plots and Egger’s test. If there was a significant publication bias, we used the “trim and fill” algorithm to correct it (Duval and Tweedie, 2000). In two-sided tests, statistical significance was defined as *p*＜0.05. The Stata 12.0 software package was used to conduct the analyses.

## 3 Results

### 3.1 Literature Search

**FIGURE 1:**
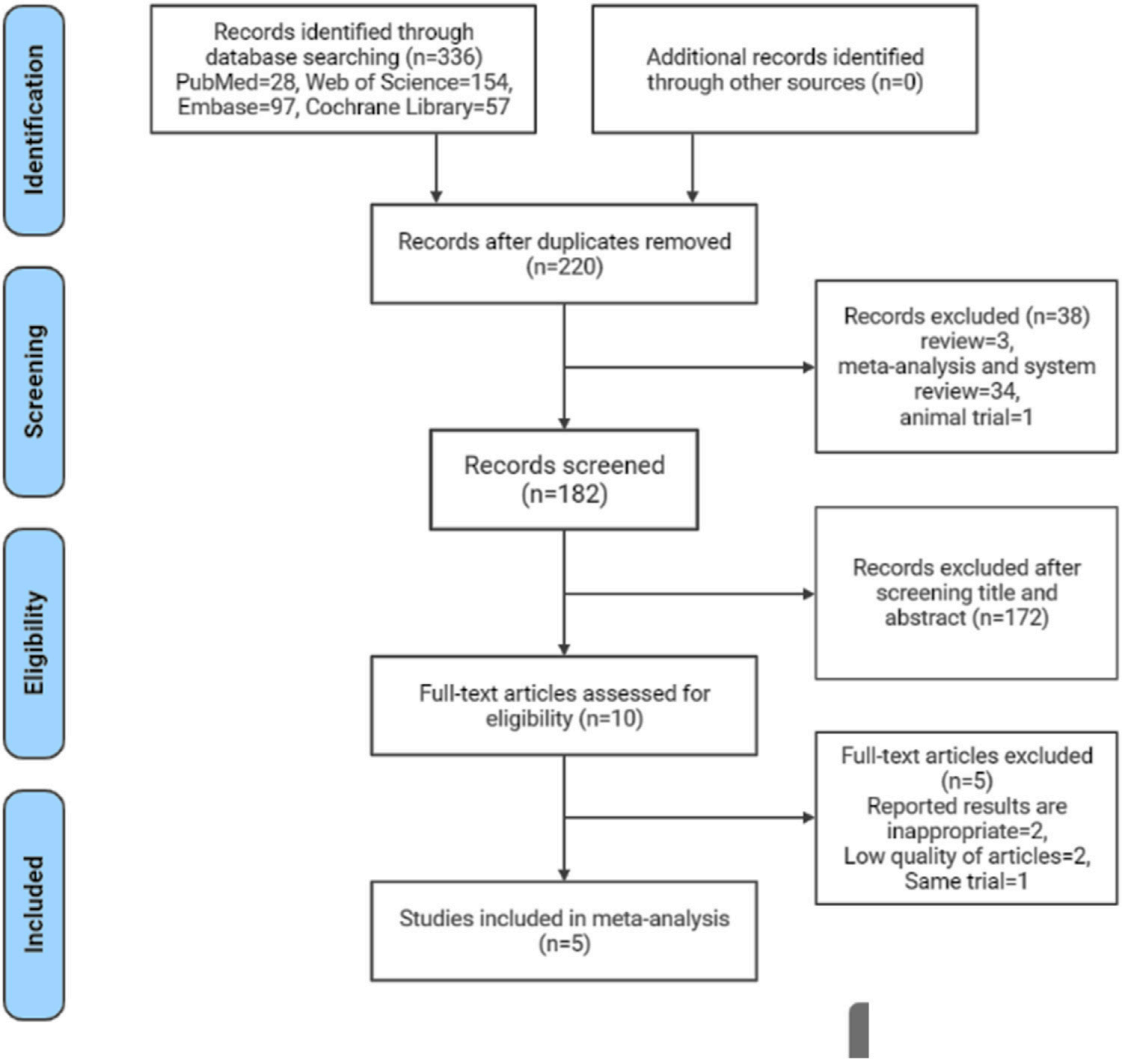
Flowchart of study selection in this systematic review and meta-analysis.

The process of literature screening, study selection, and reasons for exclusion are depicted in the PRISMA statement flowchart (Figure 1). We found 336 records in our initial search. 10 articles were considered potentially eligible for inclusion after duplicates were removed and titles and abstracts were screened. 5 RCTs (Tsai et al., 2013; Leder et al., 2015; Idolazzi et al., 2016; Nakamura et al., 2017; Suzuki et al., 2019) were finally included in the meta-analysis after a thorough review of the full text.

### 3.2 Characteristics of the Included Studies

Table 1 summarises the major characteristics of the trials included. This meta-analysis included 6 studies published between 2013 and 2018. They included 297 patients with postmenopausal osteoporosis accepting the combination treatment or monotherapy from Northern America (2 studies), Asia (2 studies), and Europe (1 study). The mean age of the included participants ranges from 67 to 75 years old. Population sizes ranged from 30 to 94. Among the selected studies, 3 of 5 trials compared combination with teriparatide, 5 of 5 compared combination with denosumab. In addition, the baseline values of the continuous variables are summarized in Table 2.

**TABLE 1:** Basic characteristics of included studies.

Author, year	Country	Sample size		Mean age (year)		Interval and dose		Adjuvant	Outcome
		Combination	Comparison	Combination	Comparison	Combination	Comparison		
Leder, 2015	America	23	(1) Teriparatide:27	65.3	(1) Teriparatide:66.1	Teriparatide 20 mg daily and denosumab 60 mg every 6 months	(1) Teriparatide 20 mg daily	Calcium Vit D	Lumbar spine BMD Total hip BMD CTX Adverse events
			(2) Denosumab:27		(2) Denosumab:65.1		(2) Denosumab 60 mg every 6 months		
Nakamura, 2017	Japan	17	Denosumab:13	75.5	Denosumab:75.1	Teriparatide 20 mg daily and denosumab 60 mg every 6 months	Denosumab 60 mg every 6 months	Calcium Vit D	Lumbar spine BMD Total hip BMD Adverse events
Suzuki, 2018	Japan	17	Denosumab:20	72.2	Denosumab:74.1	Teriparatide 20 mg daily and denosumab 60 mg every 6 months	Denosumab 60 mg every 6 months	Calcium Vit D	Lumbar spine BMD Total hip BMD Adverse events
Tsai, 2013	America	30	(1) Teriparatide:31	65.9	(1) Teriparatide:65.5	Teriparatide 20 mg daily and denosumab 60 mg every 6 months	(1) Teriparatide 20 mg daily	Calcium Vit D	Lumbar spine BMD Total hip BMD CTX Adverse events
			(2) Denosumab:33		(2) Denosumab:66.3		(2) Denosumab 60 mg every 6 months		
Idolazzi, 2016	Italy	19	(1) Teriparatide:20	78.0	(1) Teriparatide:76.0	Teriparatide 20 mg daily and denosumab 60 mg every 6 months (teriparatide added to denosumab initiated 3 months earlier)	(1) Teriparatide 20 mg daily	Calcium Vit D	Lumbar spine BMD Total hip BMD CTX
			(2) Denosumab:20		(2) Denosumab:76.0		(2) Denosumab 60 mg every 6 months		

BMD, Bone Mineral Density (areal or volumetric); CTX, Serum *β*-C-terminal telopeptide of type 1 collagen.

**TABLE 2:** Baseline clinical characteristics of included study subjects.

**Author, year**	Groups	Age (year)	Lumbar spine BMD (g/cm^2^)	Lumbar spine BMD (T score)	Total hip BMD (g/cm^2^)	Total hip BMD (g/cm^2^ or T score)	CTX (ng/ml)	1,25(OH)2D3 (pg/ml)	PTH (pg/ml)
Leder, 2015	Combination	65.300 ± 8.000	0.847 ± 0.130	—	0.750 ± 0.068	—	0.440 ± 0.170	—	—
	Teriparatide	66.100 ± 7.900	0.815 ± 0.109	—	0.756 ± 0.072	—	0.340 ± 0.150	—	—
	Denosumab	65.100 ± 6.200	0.863 ± 0.096	—	0.759 ± 0.102	—	0.410 ± 0.220	—	—
Nakamura, 2017	Combination	75.500 ± 1.400	0.730 ± 0.108	—	0.640 ± 0.108	—	—	58.200 ± 17.729	28.700 ± 14.019
	Denosumab	75.100 ± 1.800	0.799 ± 0.124	—	0.620 ± 0.041	—	—	56.100 ± 20.191	35.700 ± 10.100
Suzuki, 2018	Combination	72.200 ± 2.500	0.752 ± 0.124	—	0.601 ± 0.082	—	—	57.700 ± 25.563	28.400 ± 22.625
	Denosumab	74.100 ± 2.000	0.800 ± 0.134	—	0.631 ± 0.089	—	—	54.500 ± 28.622	32.600 ± 16.994
Tsai, 2013	Combination	65.900 ± 9.000	0.856 ± 0.131	—	0.642 ± 0.067	—	0.430 ± 0.170	—	—
	Teriparatide	65.500 ± 7.900	0.823 ± 0.111	—	0.643 ± 0.061	—	0.360 ± 0.150	—	—
	Denosumab	66.300 ± 8.300	0.866 ± 0.088	—	0.641 ± 0.086	—	0.390 ± 0.210	—	—
Idolazzi, 2016	Combination	78.000 ± 5.000	—	−3.200 ± 0.400	—	−2.000 ± 0.700	0.400 ± 0.180	—	—
	Teriparatide	76.000 ± 5.000	—	−3.400 ± 0.800	—	−2.000 ± 0.900	0.460 ± 0.200	—	—
	Denosumab	—	—	−3.400 ± 0.400	—	−1.900 ± 0.800	0.470 ± 0.210	—	—

Data are mean ± SD; BMD, Bone Mineral Density (areal or volumetric); CTX, Serum *β*-C-terminal telopeptide of type 1 collagen; PTH, Parathyroid Hormone.

### 3.3 Quality Assessment

In 5 trials, an sufficient randomized sequence was generated, and 2 trials reported proper allocation concealment. In 5 trials, participant and personnel blinding was unclear or rarely reported. The blinding of outcome assessment was unclear in 2 trials and the other 3 trials were sorted as being at low risk. None of the 5 trials had incomplete outcome data, selective reporting, or other bias (Supplementary Figures S1, S2).

### 3.4 Primary Outcome

#### 3.4.1 Mean Percent Change of BMD in Lumbar Spine

**FIGURE 2:**
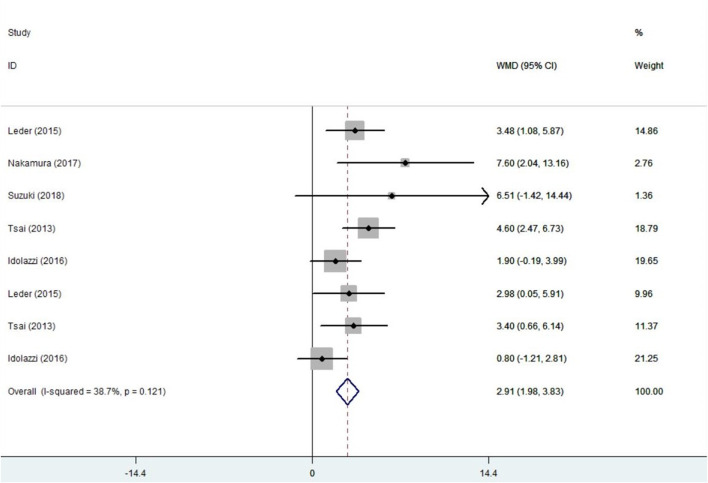
Forest plot for the lumbar spine BMD changes.

5 trials (Tsai et al., 2013; Leder et al., 2015; Idolazzi et al., 2016; Nakamura et al., 2017; Suzuki et al., 2019) with 297 patients provided the BMD data and were included in the analysis. Before conducting the meta-analysis, it is important to ensure that there is no difference between the baseline values of the combination therapy and control therapy. The positive values indicates that the combination group is higher. The results are as follows：standard mean difference (SMD) =0.05, 95%CI: −0.16∼0.26; I^2^ = 23.4%, *p* = 0.243; Z = 0.47, *p* = 0.637 (Supplementary Figure S3). There was no significant difference in baseline values between groups. Compared with the monotherapy, combination treatment can significantly improve the BMD in the lumbar spine. The results are as follows：WMD = 2.91, 95%CI: 1.98∼3.83; I^2^ = 38.7%, *p* = 0.121; Z = 6.16, *p* = 0.00 (Figure 2).

#### 3.4.2 Mean Percent Change of BMD in Hip

**FIGURE 3:**
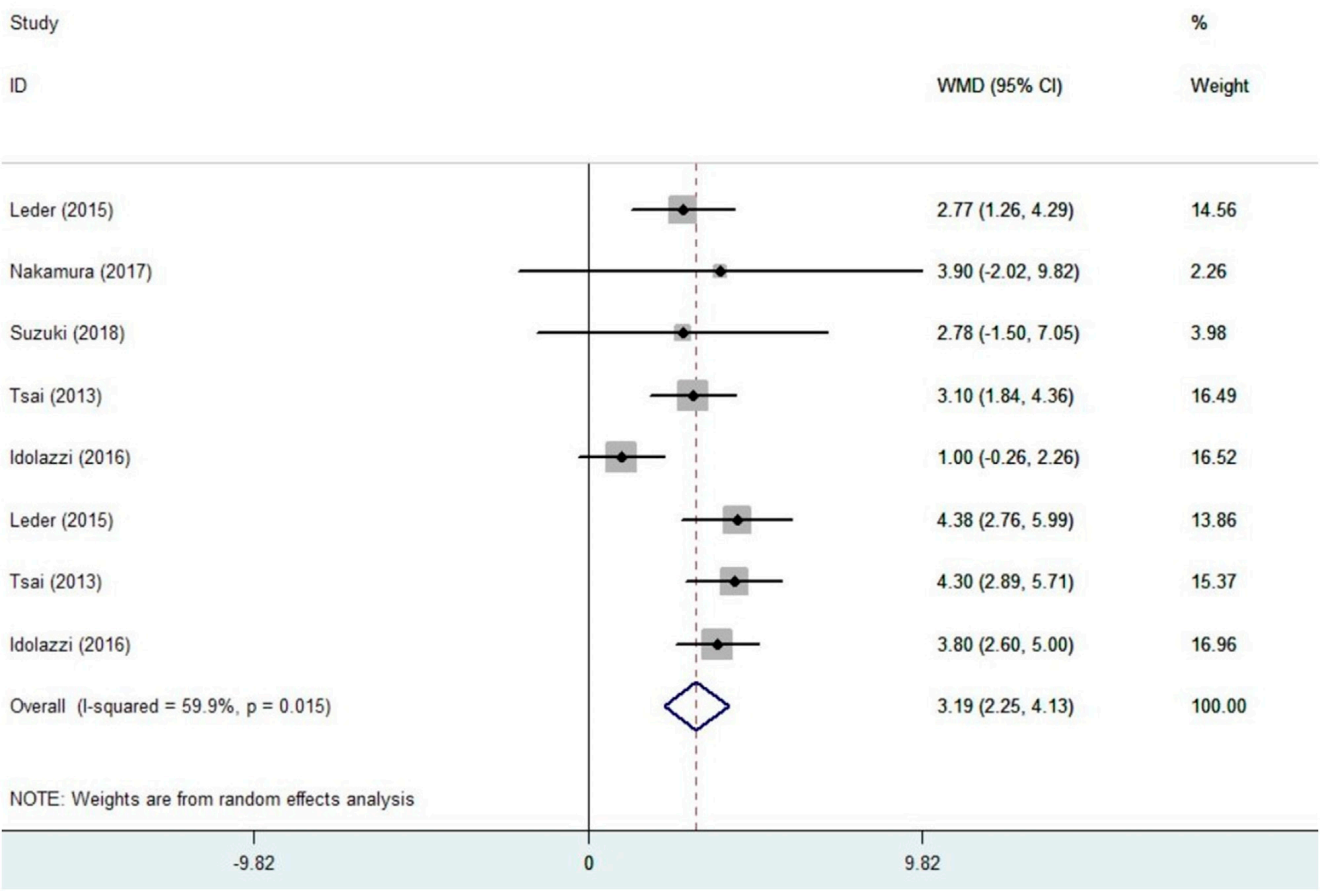
Forest plot for the total hip BMD changes.

5 trials (Tsai et al., 2013; Leder et al., 2015; Idolazzi et al., 2016; Nakamura et al., 2017; Suzuki et al., 2019) with 297 patients provided the BMD data and were included in the analysis. Before conducting the meta-analysis, it is important to ensure that there is no difference between the baseline values of the combination therapy and control therapy. The positive values indicates that the combination group is higher. The results are as follows：SMD = −0.10, 95%CI: −0.31∼0.10; I^2^ = 0.0%, *p* = 0.993; Z = 0.99, *p* = 0.322 (Supplementary Figure S4). There was no significant difference in baseline values between groups. Compared with the monotherapy, combination treatment can significantly improve the BMD in hip. The results are as follows：WMD = 3.19, 95%CI: 2.25∼4.13; I^2^ = 59.9%, *p* = 0.015; Z = 6.68, *p* = 0.00 (Figure 3).

#### 3.4.3 Incidence of Adverse Event

**FIGURE 4:**
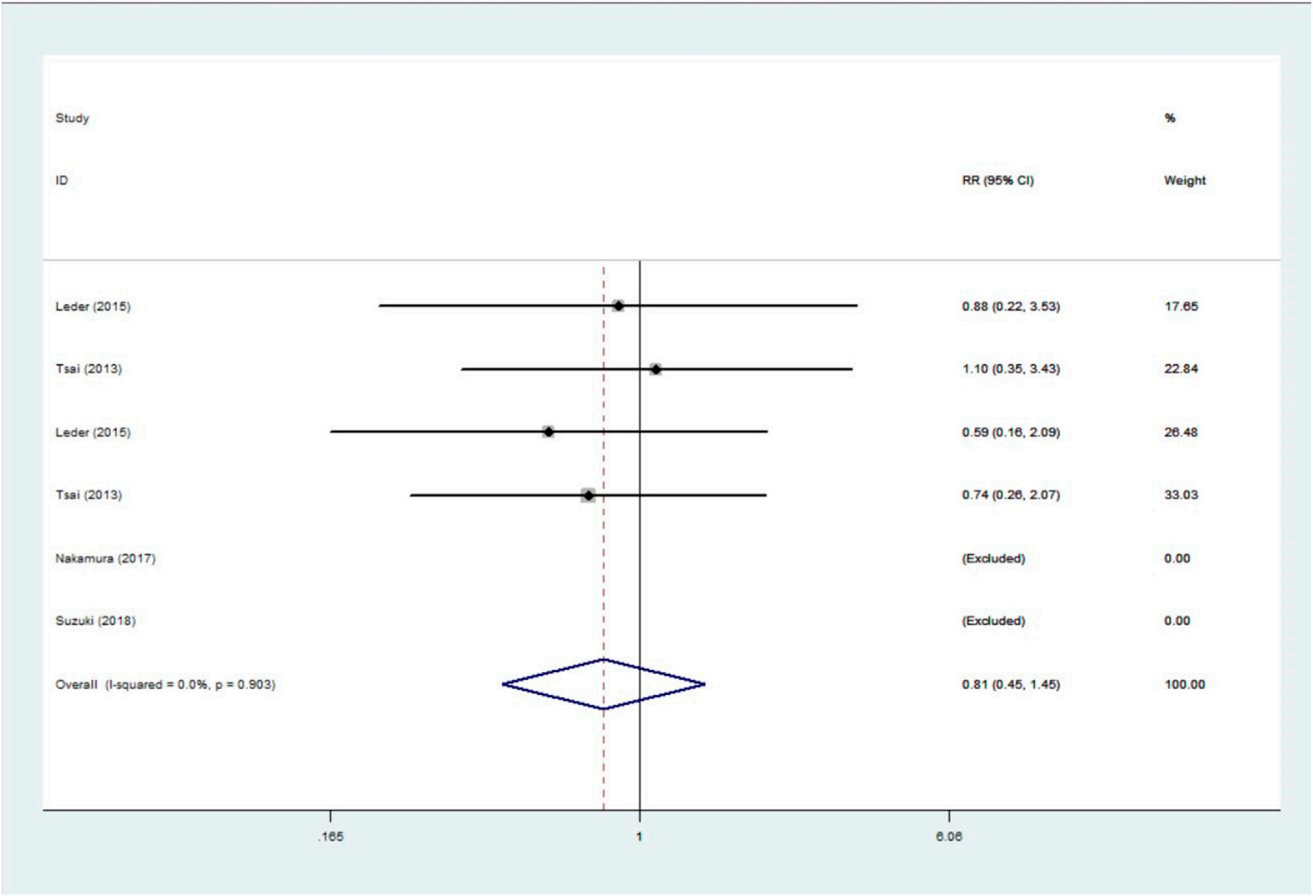
Forest plot for incidence of adverse event with combination treatment versus monotherapy.

This analysis involved 4 trials (Tsai et al., 2013; Leder et al., 2015; Nakamura et al., 2017; Suzuki et al., 2019) with a total of 238 patients. The results showed that the incidence of adverse events in the combination group was only 81% of that of the monotherapy but was not statistically significant: RR = 0.81, 95%CI: 0.45∼1.45; I^2^ = 0.0%, *p* = 0.903; Z = 0.72, *p* = 0.472 (Figure 4).

### 3.5 Second Outcome

#### 3.5.1 Mean Percent Change of CTX in Serum

3 trials (Tsai et al., 2013; Leder et al., 2015; Idolazzi et al., 2016) with 230 patients provided the CTX data and were included in the analysis. Before conducting the meta-analysis, it is important to ensure that there is no difference between the baseline values of the combination therapy and control therapy. The positive values indicates that the combination group is higher. The results are as follows：WMD = 0.03, 95%CI: −0.01∼0.08; I^2^ = 37.7%, *p* = 0.155; Z = 1.70, *p* = 0.09 (Supplementary Figure S5). There was no significant difference in baseline values between groups. We integrated the data and summarized them on a single graph (Supplementary Figure S6). The results are as follows. Compared with monotherapy, combination can decrease CTX levels significantly: WMD = −23.68, 95%CI: −33.82∼−13.54; I^2^ = 95.5%, *p* = 0.000; Z = 4.58, *p* = 0.361.

#### 3.5.2 Mean Percent Change of 25(OH)D in Serum

2 trials (Nakamura et al., 2017; Suzuki et al., 2019) with 67 patients provided the 25(OH)D data and were included in the analysis. Before conducting the meta-analysis, it is important to ensure that there is no difference between the baseline values of the combination therapy and control therapy. The positive values indicates that the combination group is higher. The results are as follows：WMD = 2.52, 95%CI: −8.32∼13.37; I^2^ = 0.0%, *p* = 0.923; Z = 0.46, *p* = 0.648 (Supplementary Figure S7). There was no significant difference in baseline values between groups. Compared with denosumab, combination can improve 25(OH)D levels but has no statistical significance: WMD = 12.27, 95%CI: −8.91∼33.45; I^2^ = 27.2%, *p* = 0.241; Z = 1.14, *p* = 0.256 (Supplementary Figure S8).

#### 3.5.3 Mean Percent Change of PTH in Serum

2 trials (Nakamura et al., 2017; Suzuki et al., 2019) with 67 patients provided the PTH data and were included in the analysis. Before conducting the meta-analysis, it is important to ensure that there is no difference between the baseline values of the combination therapy and control therapy. The positive values indicates that the combination group is higher. The results are as follows：WMD = −6.15, 95%CI: −13.36∼1.06; I^2^ = 0.0%, *p* = 0.726; Z = 1.67, *p* = 0.094 (Supplementary Figure S9). There was no significant difference in baseline values between groups. Compared with denosumab, combination can significantly improve PTH levels: WMD = 22.28, 95%CI: 2.17∼42.39; I^2^ = 0.0%, *p* = 0.876; Z = 2.17, *p* = 0.03 (Supplementary Figure S10).

### 3.6 Subgroup Analysis

#### 3.6.1 Subgroup Analysis of Mean Percent Change of BMD in Lumbar Spine

Although the heterogeneity among the five trials (Tsai et al., 2013; Leder et al., 2015; Idolazzi et al., 2016; Nakamura et al., 2017; Suzuki et al., 2019) included in this meta-analysis was low (I^2^ = 38.7%), we still perform subgroup analyses based on different control measures to determine the difference between combination treatment and specific monotherapy. Patients in the combination group had a statistically significant 3.57% increase in lumbar BMD compared to patients in the denosumab alone group. Similarly, patients in the combination group had a statistically significant 2.00% increase in lumbar BMD compared to patients in the teriparatide alone group. The specific results are shown in Table 3.

**TABLE 3:** Subgroup analysis.

Subgroup	WMD (95% CI)	I^2^ (%)	*p* value
Mean percent change of BMD in lumbar spine			
Combination therapy vs. denosumab monotherapy	3.57 (2.35, 4.79)	32.30	0.000
Combination therapy vs. teriparatide monotherapy	2.00 (0.59, 3.42)	28.70	0.006
Mean percent change of BMD in hip			
Combination therapy vs. denosumab monotherapy	2.28 (1.53, 3.03)	36.90	0.000
Combination therapy vs. teriparatide monotherapy	4.10 (3.30, 4.90)	0.00	0.000
Mean percent change of CTX in serum			
Combination therapy vs. denosumab monotherapy	3.19 (−8.32, 14.71)	87.50	0.587
Combination therapy vs. teriparatide monotherapy	−116.50 (−137.90, −95.11)	0.00	0.000

BMD, Bone Mineral Density; CTX, Serum *β*-C-terminal telopeptide of type 1 collagen; CI, Confidence Interval.

#### 3.6.2 Subgroup Analysis of Mean Percent Change of BMD in Hip

Since there was moderate heterogeneity (I^2^ = 59.9%) among the 5 trials (Tsai et al., 2013; Leder et al., 2015; Idolazzi et al., 2016; Nakamura et al., 2017; Suzuki et al., 2019) included in this meta-analysis, we took a subgroup analysis to explore the sources of heterogeneity. Meta-regression analyses found a significant effect of control measures (*t* = 2.57, *p* = 0.042) on the mean percent change of BMD in hip. Based on this, we performed subgroup analyses based on different control measures and found the heterogeneity decreases significantly. The combined results suggest that patients in the combination group had a statistically significant 2.28% increase in hip BMD compared to patients in the denosumab alone group. Similarly, patients in the combination group had a statistically significant 4.10% increase in hip BMD compared to patients in the teriparatide alone group. The specific results are shown in Table 3.

#### 3.6.3 Subgroup Analysis of Mean Percent Change of CTX in Serum

Since there was strong heterogeneity (I^2^ = 95.5%) among the 3 trials (Tsai et al., 2013; Leder et al., 2015; Idolazzi et al., 2016) included in this meta-analysis compared combination with denosumab, we took a subgroup analysis to explore the sources of heterogeneity. Based on this, we performed subgroup analyses based on different control measures. Compared with denosumab, combination can improve CTX levels but has no statistical significance: WMD = 3.19, 95%CI: −8.32∼14.71; I^2^ = 87.5%, *p* = 0.000; Z = 0.54, *p* = 0.587; compared with teriparatide, combination can significantly decrease CTX levels: WMD = −116.50, 95%CI: −137.90∼−95.11; I^2^ = 0.0%, *p* = 0.385; Z = 10.67, *p* = 0.00. The specific results are shown in Table 3.

### 3.7 Sensitivity Analysis

To test the results’ stability, a sensitivity analysis was carried out. In this meta-analysis, the mean percent change of BMD in hip remained stable and constant after each study was removed one at a time (Supplementary Figure S11).

### 3.8 Publication Bias

#### 3.8.1 Mean Percent Change of BMD in Lumbar Spine

To examine the publication bias of the 5 trials (Tsai et al., 2013; Leder et al., 2015; Idolazzi et al., 2016; Nakamura et al., 2017; Suzuki et al., 2019) in this meta-analysis, the following funnel plots were drawn (Supplementary Figure S12). Further, the Eegg’s test yielded *p* = 0.114 > 0.05. Therefore, there was no publication bias in the current study.

#### 3.8.2 Mean Percent Change of BMD in Hip

To examine the publication bias of the 5 trials (Tsai et al., 2013; Leder et al., 2015; Idolazzi et al., 2016; Nakamura et al., 2017; Suzuki et al., 2019) in this meta-analysis, the following funnel plots were drawn (Supplementary Figure S13). Further, the Eegg’s test yielded *p* = 0.807 > 0.05. Therefore, there was no publication bias in the current study.

## 4 Discussion

We discovered in this meta-analysis of 6 RCTs that the combination of teriparatide and denosumab was superior to monotherapy with these two drugs in improving BMD in lumbar spine and hip for patients with postmenopausal osteoporosis. This result is congruent with a recently published meta-analysis by Lou et al. (Lou et al., 2019). However, in Lou’s meta-analysis, the subjects were osteoporosis rather than postmenopausal osteoporosis patients, and the interventions were similarly expanded to combination treatment with parathyroid peptide analogues and antiresorptive agents. Considering the differences between the 2 meta-analyses, we compared the results with caution. Besides, to clarify the specific differences between combination treatment and denosumab or teriparatide, we performed further subgroup analyses. We found that the combination group could improve lumbar spine BMD and hip BMD by 3.57 and 2.0%, respectively, compared with denosumab. Compared with teriparatide, the combination group could improve 2.28 and 4.10%, respectively. Interestingly, the comparison of the combination group with denosumab or teriparatide showed opposite results in the meta-analysis of CTX. The combination group was elevated by 16.49% compared to denosumab and decreased by 116.50% compared to teriparatide. This phenomenon may be associated with the mechanism of action of both medicines. Denosumab inhibits bone resorption by antagonizing osteoclasts but teriparatide promotes bone resorption at the same time as it promotes bone synthesis (Cummings et al., 2009; Tsai et al., 2019). In terms of safety, this meta-analysis showed that the incidence of adverse events in the combination group was only 82% of that of the monotherapy modalities, but the results were not statistically significant. We know that the side effect of teriparatide is to increase serum calcium while causing dizziness, poor appetite, muscle, and joint pain (Langdahl et al., 2017), while the side effect of denosumab is to reduce blood calcium (Lecoq et al., 2021), if the combination of the two drugs will reduce the side effect in some aspects? This requires more research to further explore.

Osteoporotic fractures have a direct patient burden in terms of morbidity and mortality, as well as a substantial societal economic burden due to direct healthcare resource consumption, direct nonmedical expenses, and indirect expenses (Burge et al., 2007; Pike et al., 2011; Bliuc et al., 2013; Tajeu et al., 2014). The vast majority of patients suffering from osteoporosis are postmenopausal women (Selby, 2004). Bisphosphonates are recommended as the first line of medication for postmenopausal osteoporosis, but there are growing concerns about their increasingly obvious side effects (Black and Rosen, 2016b; Vargas-Franco et al., 2018). Along with clinical trials, teriparatide and denosumab are gradually gaining public recognition for their use in postmenopausal osteoporosis (Chen et al., 2006; Cummings et al., 2009). In particular, attention has been drawn to the efficacy produced by the combination of the two drugs. The combination of teriparatide and denosumab, on the other hand, enhanced lumbar spine and hip BMD more than either drug alone, and to a greater extent than any of the currently available drugs (Tsai et al., 2013). The capability of denosumab to completely inhibit teriparatide-induced bone resorption but only partially inhibit teriparatide-induced bone formation seems to be the cause of the superimposed effect (Tsai et al., 2013). Denosumab could regulate the canonical wnt signaling pathway by upregulating sclerostin and inhibiting DKK1 expression (Glass et al., 2005; Diarra et al., 2007; Gatti et al., 2012). This mechanism may have important implications for the regulation of bone metabolism during combination drug administration. These findings are consistent with findings from other animal models, such as the ovariectomized rat, in which the combination of osteoprotegerin (an innate chemical with properties similar to denosumab) and teriparatide greatly reduced osteoclast quantity, did not decrease osteoblast quantity, and improved trabecular and cortical BMD more than either agent alone (Kostenuik et al., 2001). Correspondingly, in a mouse model where osteoprotegerin and teriparatide were co-administered, the improvement in femoral BMD was additive, and the improvement in spine BMD outweighed the cumulative change with each agent alone (Samadfam et al., 2007). While the detailed mechanisms have not been illuminated, these findings demonstrate the significant efficacy of the combination of teriparatide and denosumab in postmenopausal osteoporosis.

Our meta-analysis has several strengths. Previously, there was a meta-review on the combination treatment of parathyroid peptide analogues and antiresorptive medications for osteoporosis. However, this meta-analysis is the first to review the combination of teriparatide and denosumab in postmenopausal osteoporosis. Second, the quality of RCTs included in this meta-analysis was generally high. Third, although there was certain heterogeneity in some results, we reduced or even eliminated the heterogeneity by conducting subgroup analyses with various stratification factors. On the other hand, our meta-analysis also has several limitations. First, the original studies had methodological flaws, such as ambiguous randomization methods and insufficient treatment allocation concealment. Second, the sample size was relatively small in our meta-analysis. Third, with the language limited to English, some potential studies may be missed.

## 5 Conclusion

The meta-analysis indicates that combination treatment led to greater BMD at the lumbar spine and hip in comparison to monotherapy, without an increased incidence of adverse events.

## Data Availability

The original contributions presented in the study are included in the article/Supplementary Material, further inquiries can be directed to the corresponding author.
