# Supplementary material for: Efficacy of the Combination of Teriparatide and Denosumab in the Treatment of Postmenopausal Osteoporosis: A Meta-Analysis
Source: Front Pharmacol. 2022 May 24;13:888208. doi: 10.3389/fphar.2022.888208 (PMC9170942; doi:10.3389/fphar.2022.888208)
Supplement: Supplementary file 2 [file DataSheet1.docx]

Supplementary Material

# Supplementary Figures


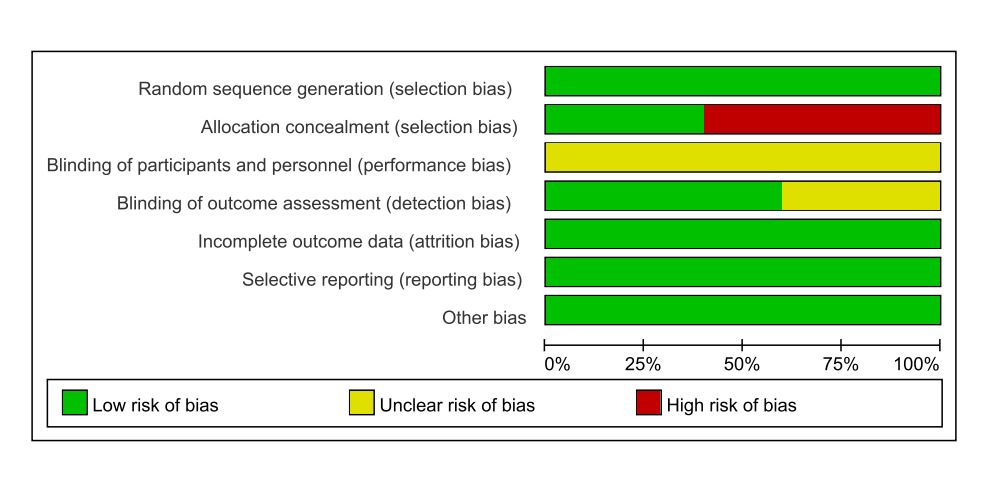


**Supplementary Figure S1.** Risk of bias graph.


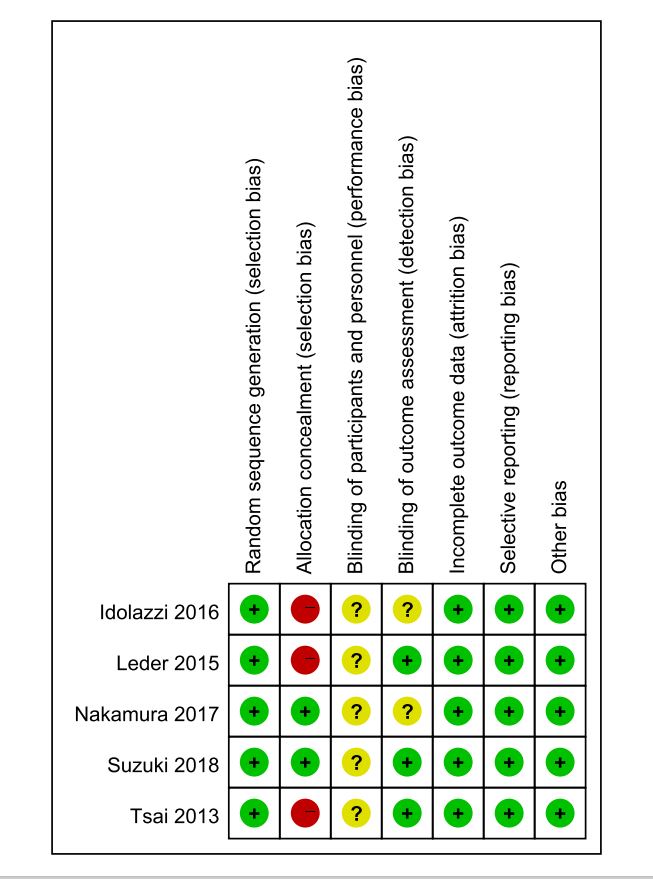


**Supplementary Figure S2.** Risk of bias summary.


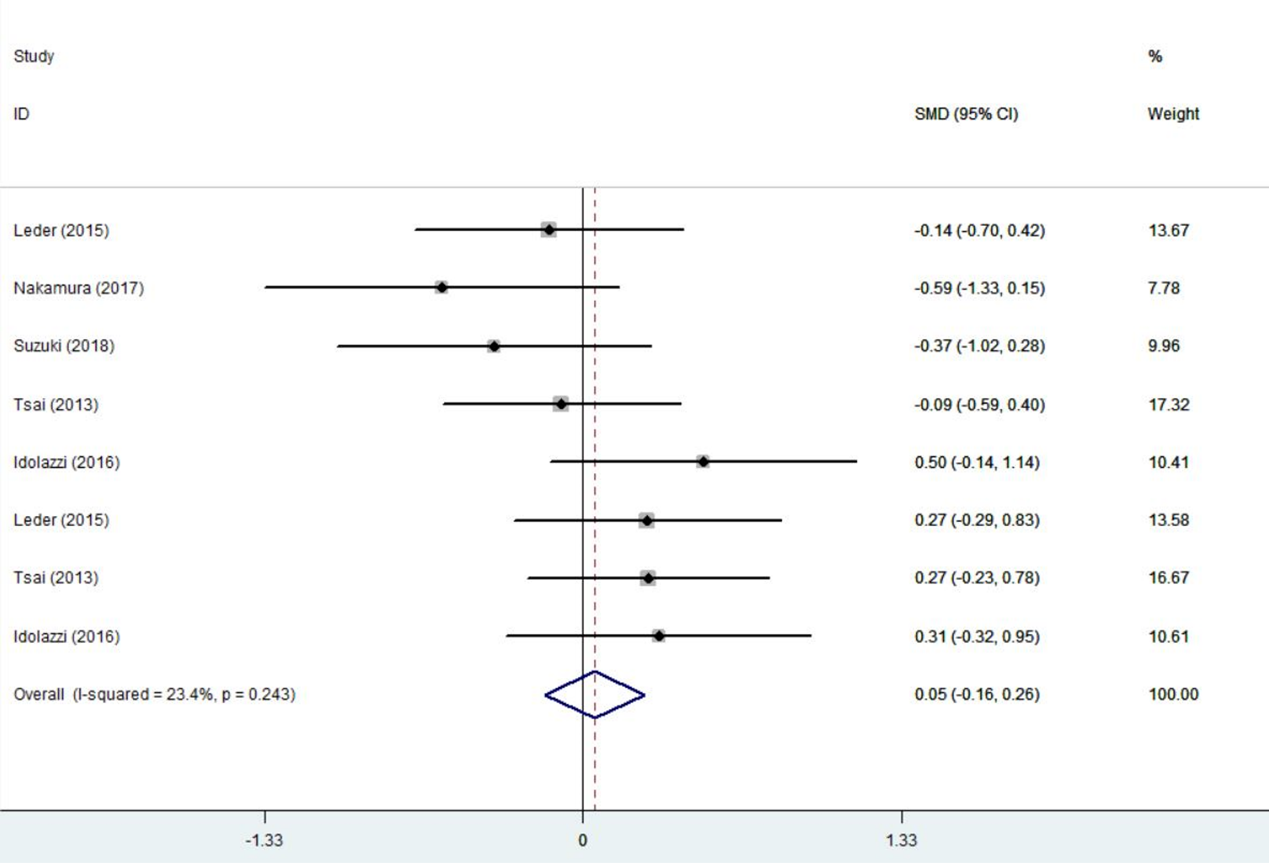


**Supplementary Figure S3.** Forest plot for the difference between the baseline values of the combination therapy and control therapy in lumbar spine BMD .


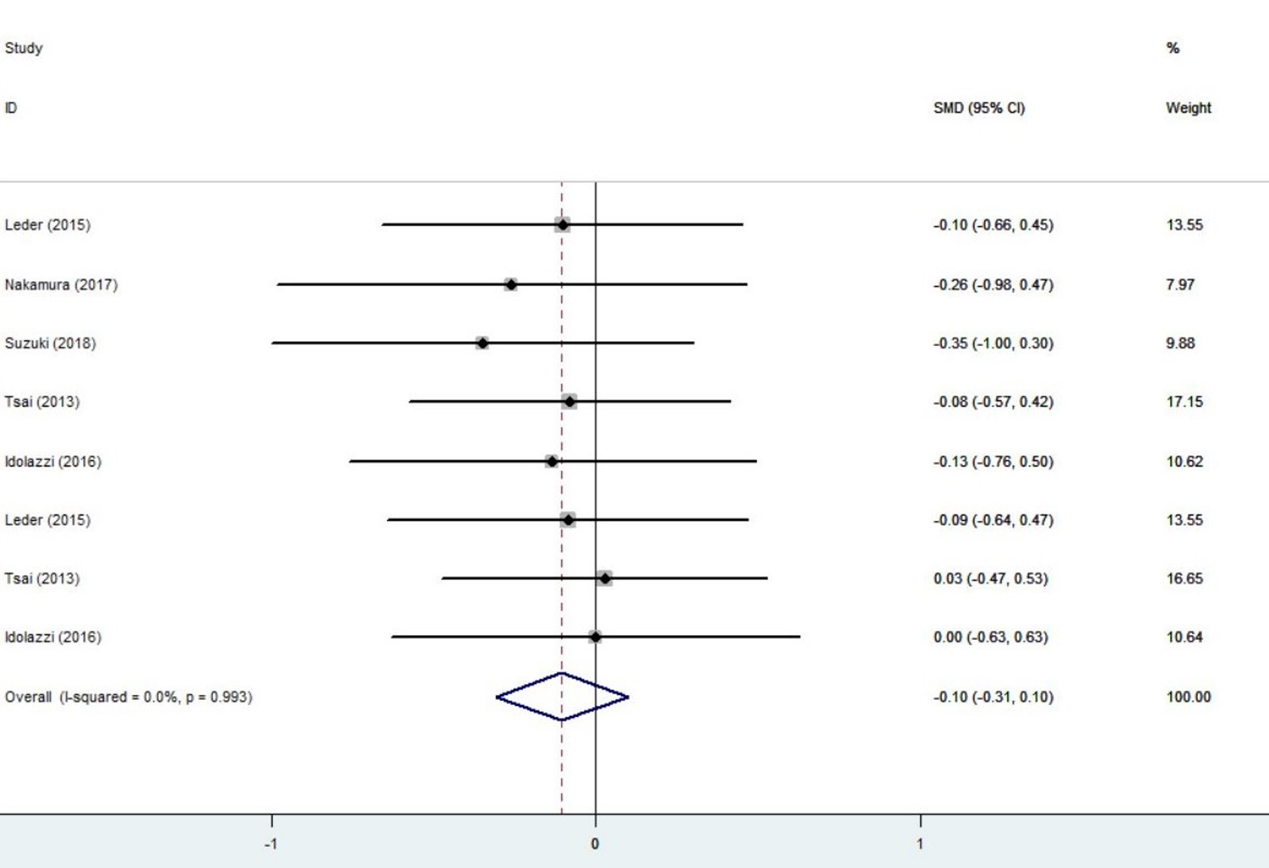


**Supplementary Figure S4.** Forest plot for the difference between the baseline values of the combination therapy and control therapy in total hip BMD.


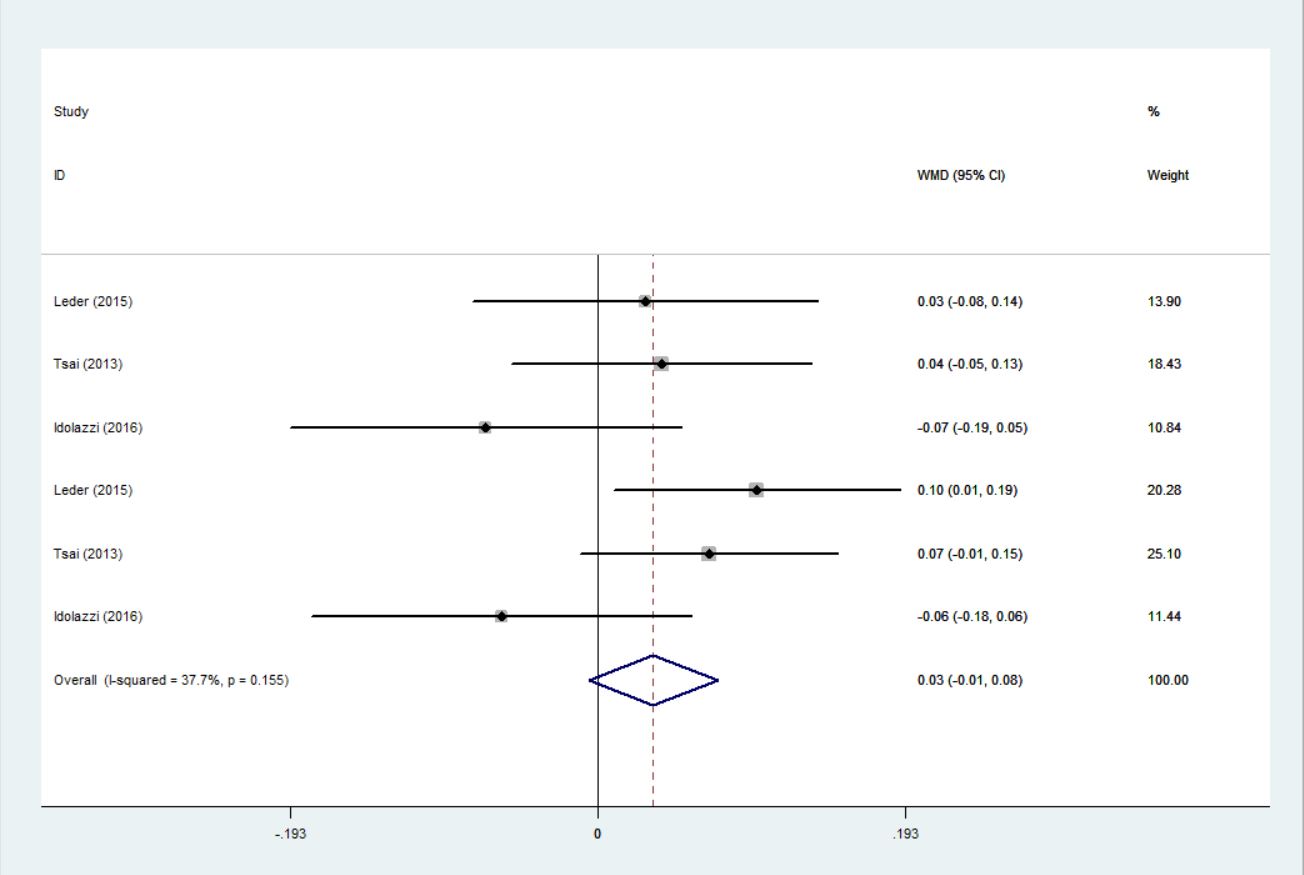


**Supplementary Figure S5.** Forest plot for the difference between the baseline values of the combination therapy and control therapy in serum CTX.


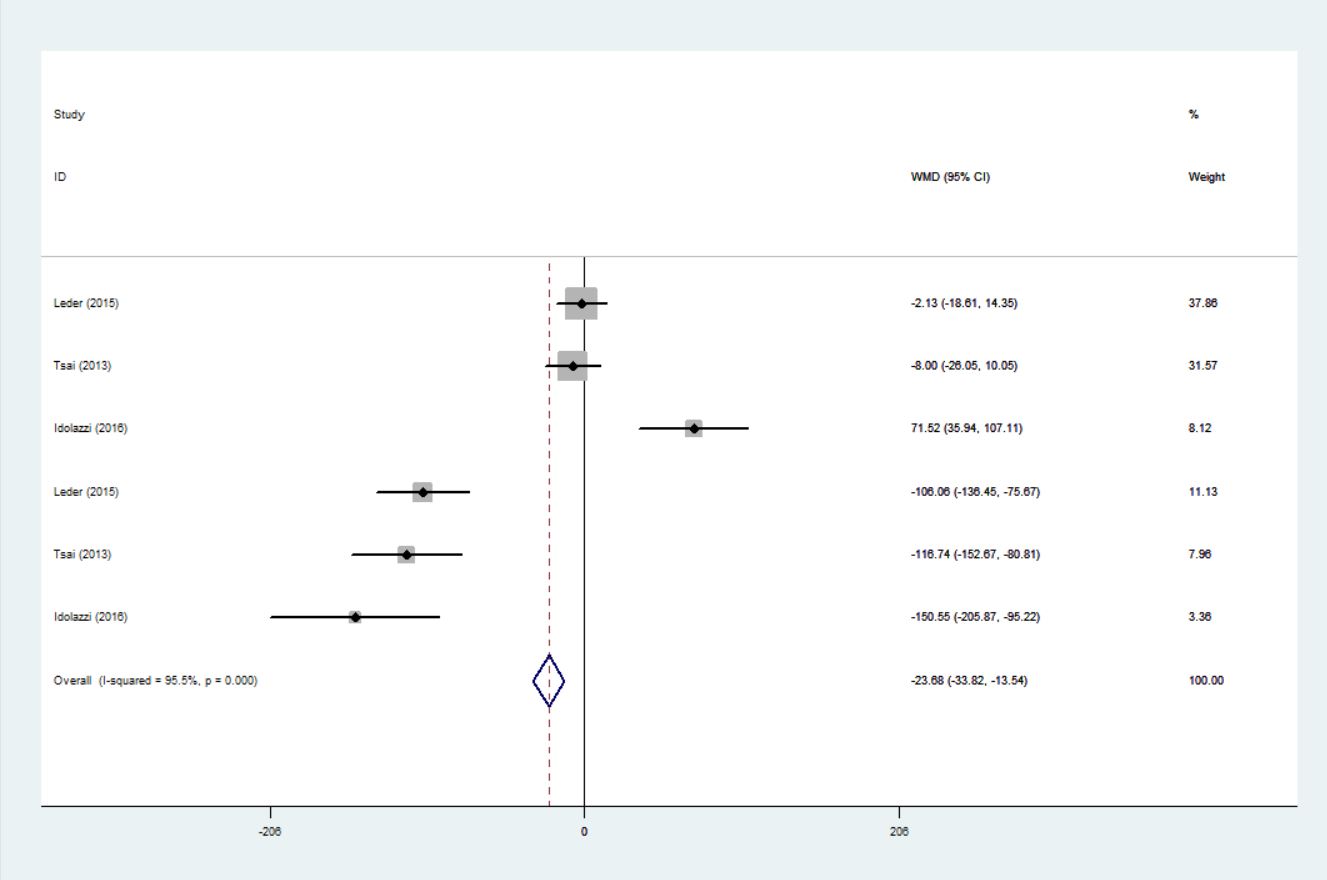


**Supplementary Figure S6.** Forest plot for the serum CTX changes


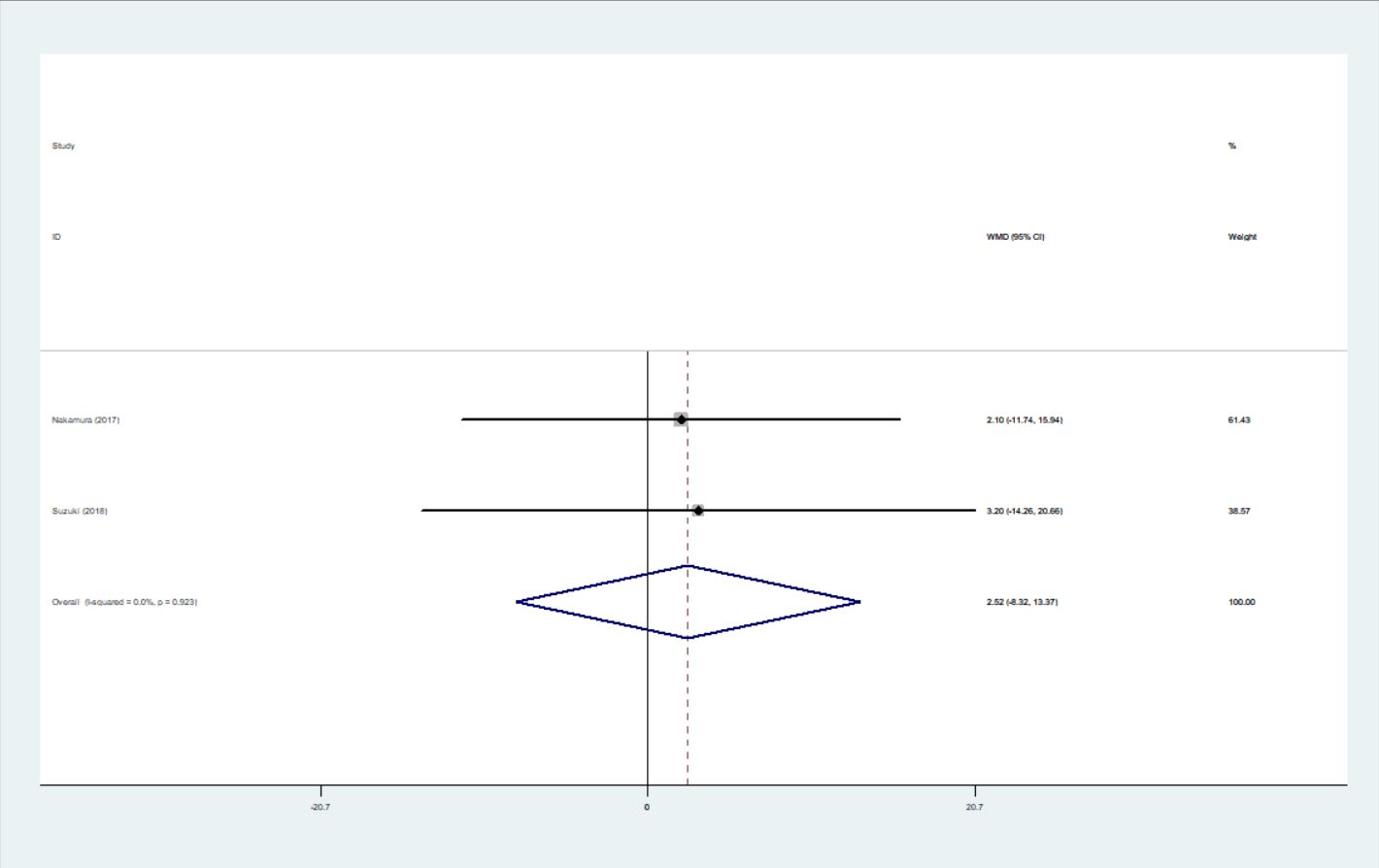


**Supplementary Figure S7.** Forest plot for the difference between the baseline values of the combination therapy and control therapy in serum 25(OH)D.


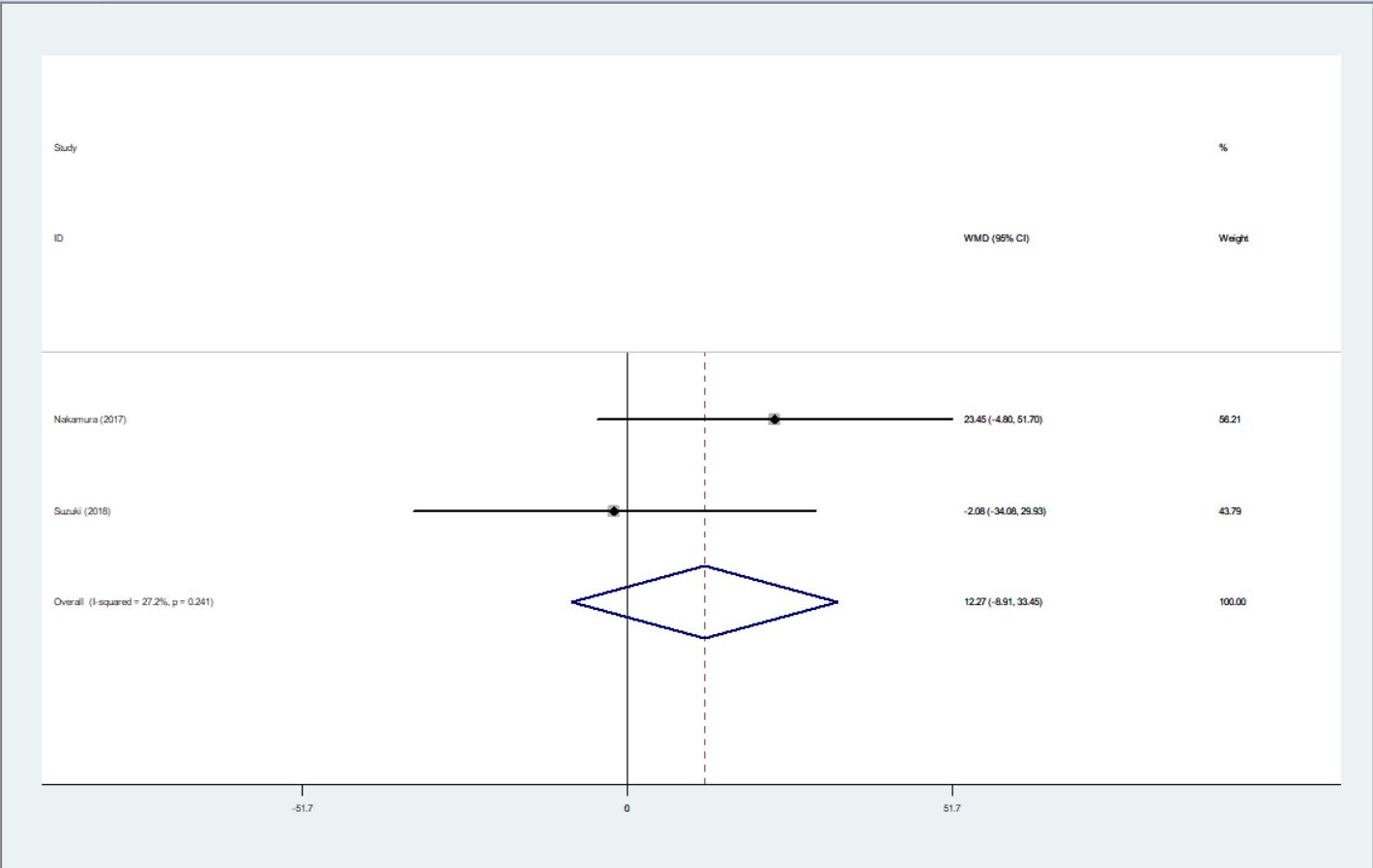


**Supplementary Figure S8.** Forest plot for the serum 25(OH)D changes


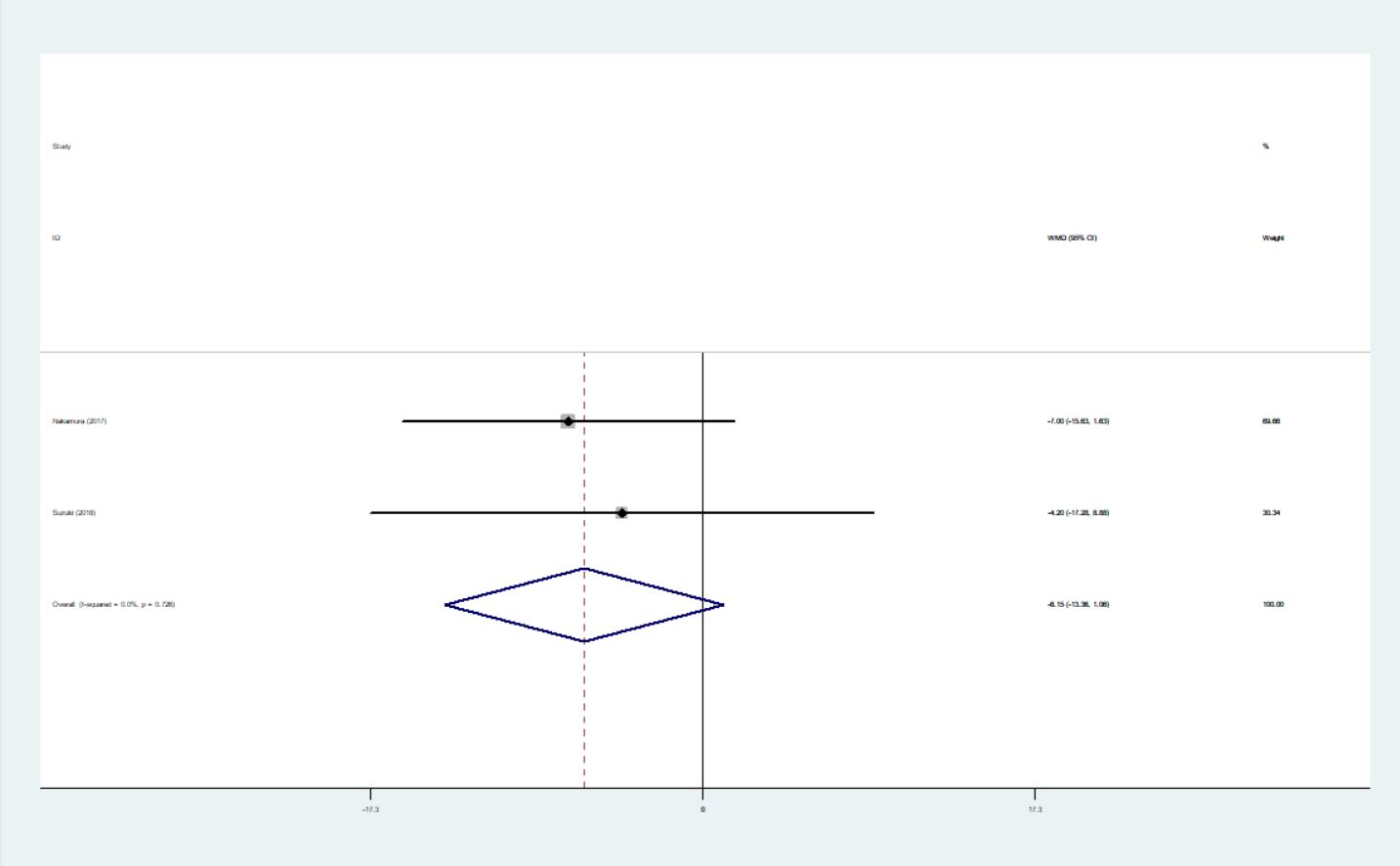


**Supplementary Figure S9.** Forest plot for the difference between the baseline values of the combination therapy and control therapy in serum PTH.


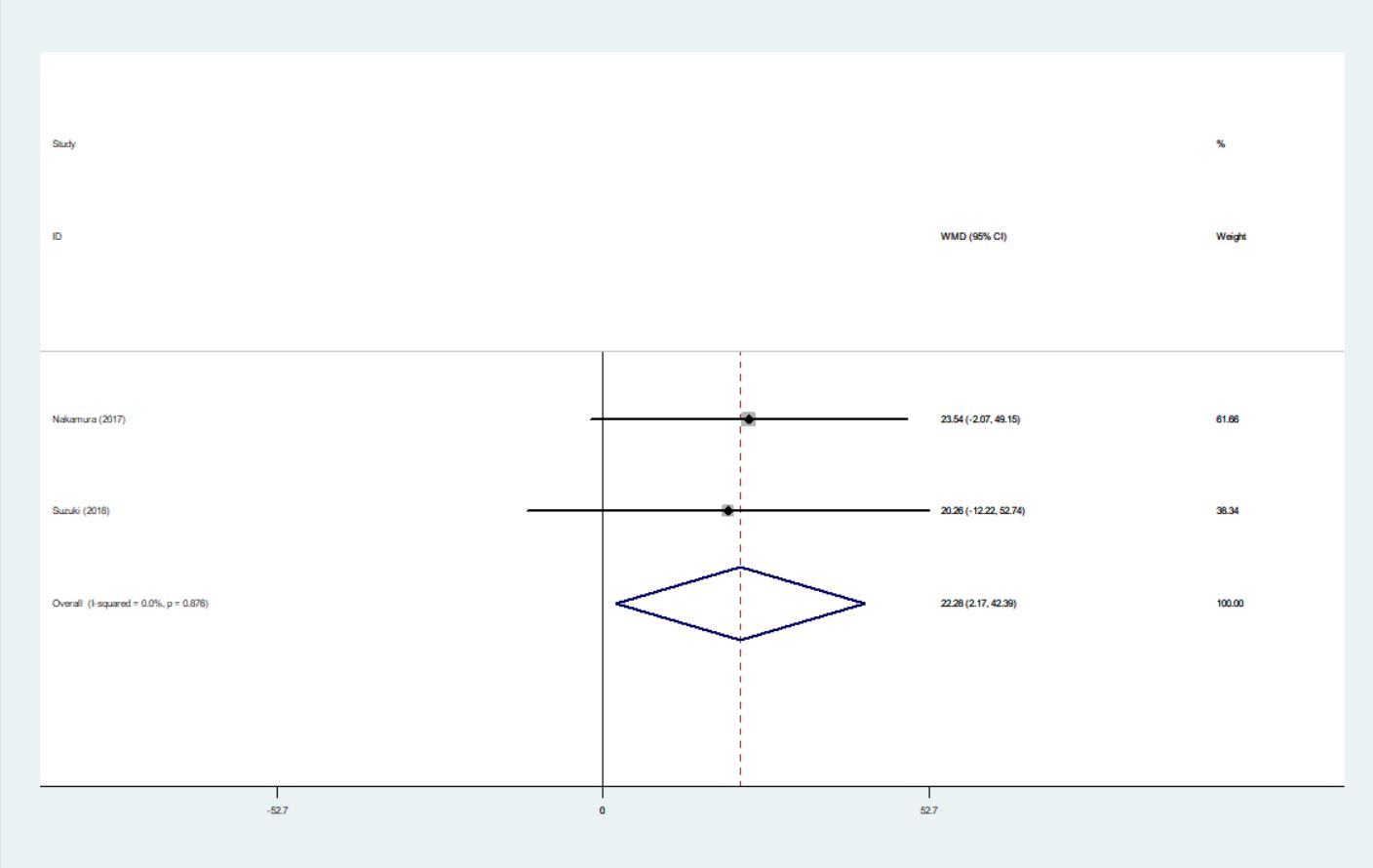


**Supplementary Figure S10.** Forest plot for the serum PTH changes


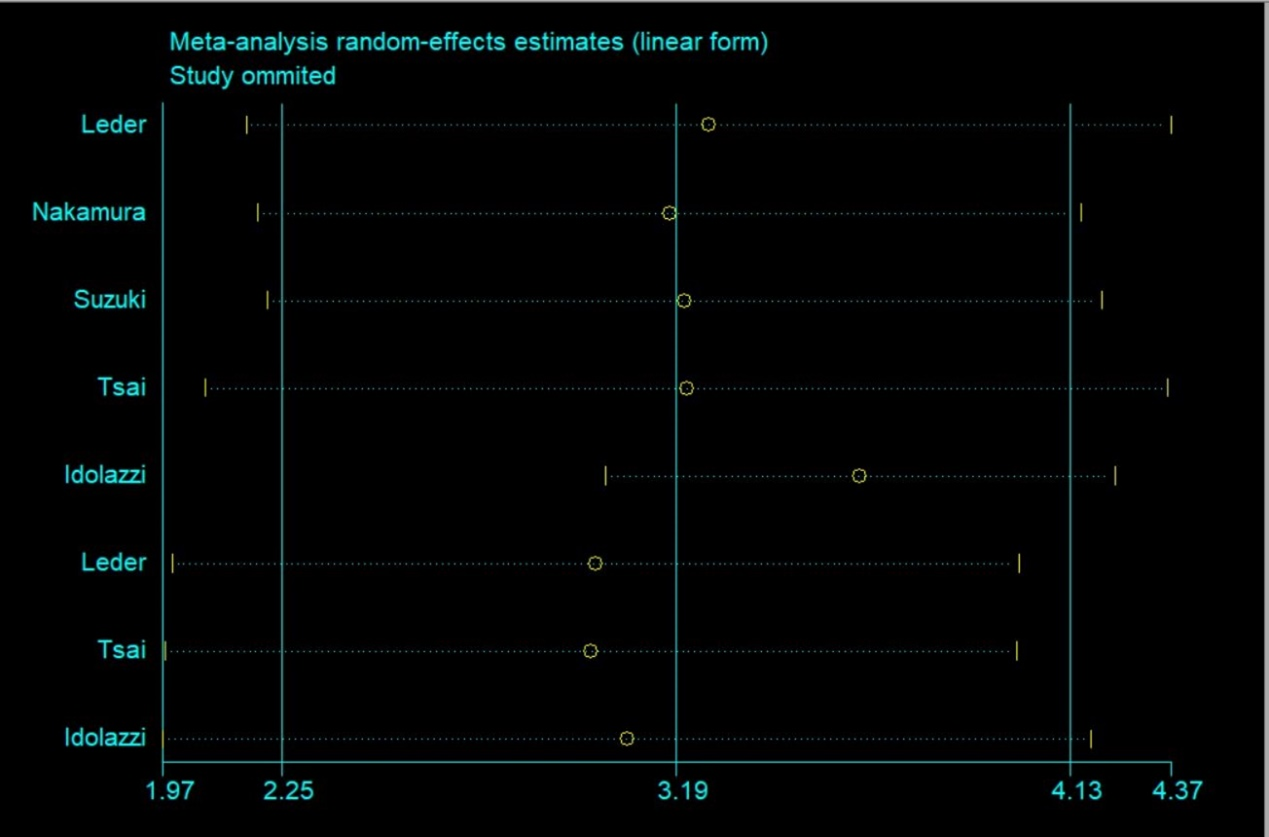


**Supplementary Figure S11.** Sensitivity analyses for mean percent change of BMD in hip.


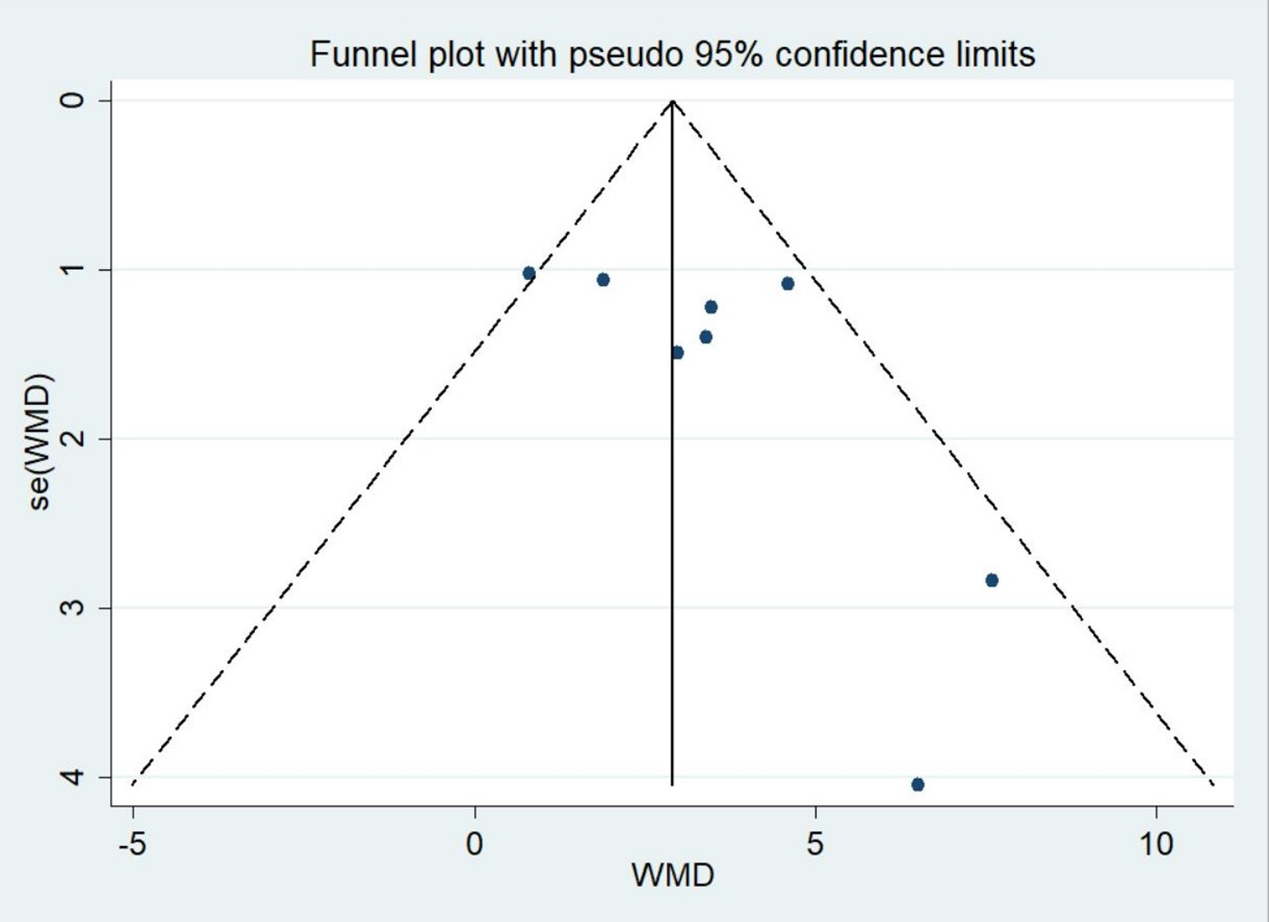


**Supplementary Figure S12.** The publication bias for mean percent change of BMD in lumbar spine.


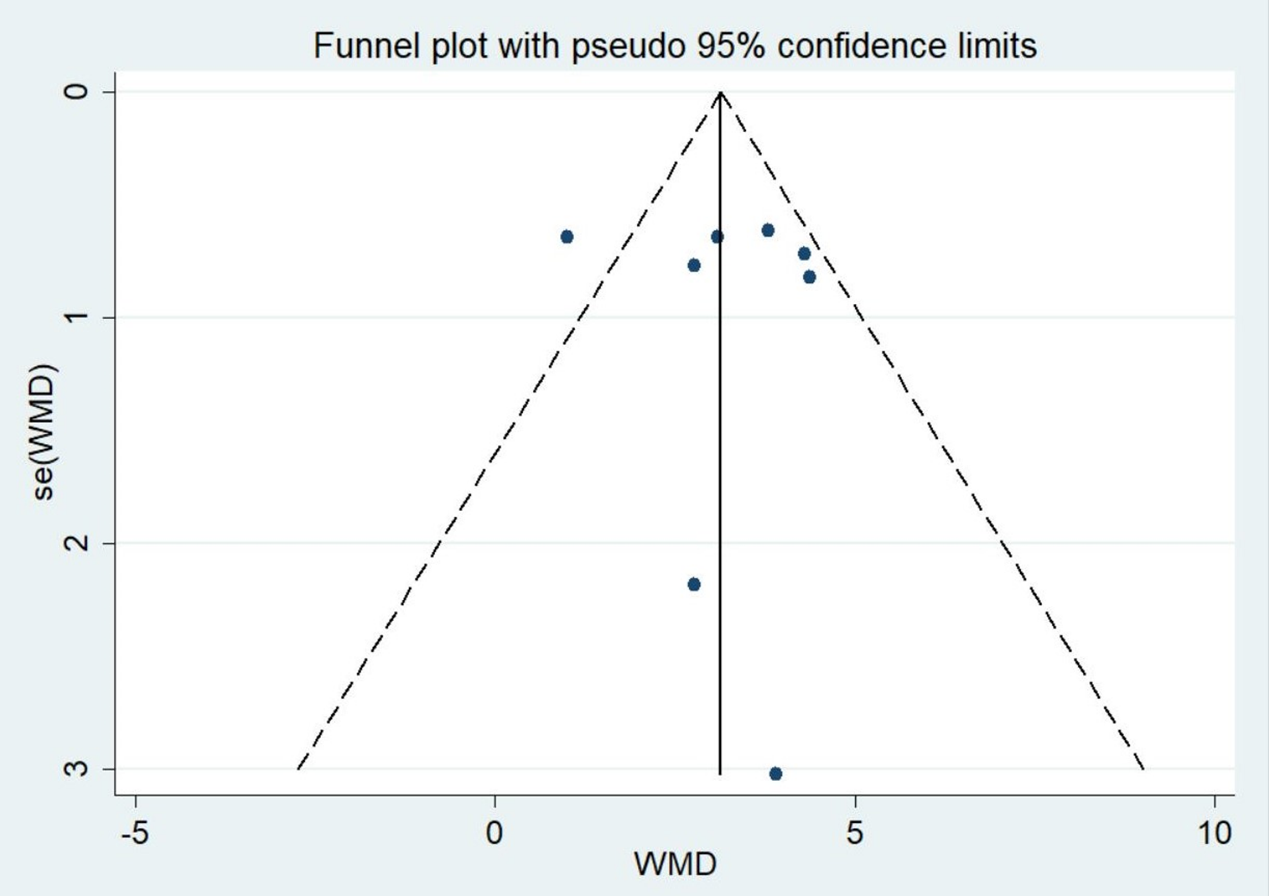


**Supplementary Figure S13.** The publication bias for mean percent change of BMD in hip
